# A review of the possibility of adopting financially driven live donor kidney transplantation

**DOI:** 10.1590/S1677-5538.IBJU.2017.0693

**Published:** 2018

**Authors:** Aline Adour Yacoubian, Rana Abu Dargham, Raja B. Khauli

**Affiliations:** 1 Department of Surgery, American University of Beirut Medical Center, Beirut, Lebanon; 2 Division of Urology and Renal Transplantation, American University of Beirut Medical Center, Beirut, Lebanon

**Keywords:** Kidney Transplantation, Kidney Diseases, Review [Publication Type]

## Abstract

Kidney transplantation for end-stage renal disease remains the preferred solution due to its survival advantage, enhanced quality of life and cost-effectiveness. The main obstacle worldwide with this modality of treatment is the scarcity of organs. The demand has always exceeded the supply resulting in different types of donations. Kidney donation includes pure living related donors, deceased donors, living unrelated donors (altruistic), paired kidney donation and more recently compensated kidney donation. Ethical considerations in live donor kidney transplantation have always created a debate especially when rewarding unrelated donors. In this paper, we examine the problems of financially driven kidney transplantation, the ethical legitimacy of this practice, and propose some innovative methods and policies that could be adopted to ensure a better practice with accepted ethical guidelines.

## INTRODUCTION

Living related kidney donation evolved significantly between 1960s and 1970s and became a routinely acceptable practice (1). With the improvements and availability of maintenance dialysis in the 1980s and 1990s, deceased donor kidney transplantation led to enhanced numbers but with limited success (1). Traditional cultural beliefs continue to persist in some countries like in China wherein dead bodies should be kept intact and no organ should be used for donation (2). The gap between supply and demand of kidneys continues to rise and is expected to rise more with a clear inconsistency between the number of transplants and the number of patients on the waiting list (3). For instance, in China, around 1.5 million Chinese patients are placed on the organ waiting list every year, while less than 1% receive an organ, because only relatives are allowed to donate (2). Absence of donors in Qatar has obliged most patients with end-stage renal disease to seek commercial donors abroad and return with high postoperative complications (4). Anecdotal evidence also shows that commercial kidney transplants take place in third world countries such as in India, Pakistan, Cambodia, Sri Lanka wherein potential recipients or patients may seek poor donors (5).

The most commonly accepted method of live kidney donation, altruism, remains insufficient since it does not help halt the illegal buying and selling of kidneys (1). Altruism occurs very rarely due to its challenges in trying to find such donors. Although many countries, like China, have initiated the deceased donor organ donation, the issue of shortage has not been solved (2). Additionally, the paucity of deceased donor organs has recently contributed to the surge of living unrelated transplants (1) as evidence shows that even if supposedly all kidneys were supplied from deceased donors, the supply would still not be enough to satisfy the increasing demand (6). However, the solution of living unrelated donation especially with commercialization has resulted in ethical dilemmas.

In 2008, the Transplantation Society in Turkey organized the International Summit on Transplant Tourism and Organ Trafficking. The Summit released the Istanbul Declaration which emphasizes the importance of preventing organ trafficking and transplant commercialism and encourages legitimate transplantation protocols (1). However, severe organ scarcity along with increasing suffering and death of patients on waiting lists have overpowered the rejection of commercialization and altruistic paradigm (7). On another note, living donation seems promising as it aims to add the number of donor organs and enhance the overall efficacy of transplants (6); it can also reduce trafficking, but waiting lists continue to grow (8). As a result, a great focus has been put on integrating financial rewards to increase the number of unrelated living donations rather than relying solely on altruistic donors.

The American Society of Transplantation's Live Donor Community of Practice organized a Consensus Conference on Best Practices in Live Kidney Donation in 2014 (9). The group generated the following guidelines: assign resources for standardized reimbursement of lost wages and incidental costs for live kidney donation; pass legislation to propose employment and insurability protections to live kidney donations; generate live kidney donation financial toolkit to deliver standardized and evaluated education to donors and providers about options to increase donor coverage and reduce financial effect within the current climate; and endorse additional research to recognize possible barriers to living donation and live kidney donation to ensure the creation of potential strategies (9).

In this review, we highlight the different types of kidney donation and emphasize the ethical dilemmas in financial rewards for living kidney donors, and discuss the reasons for the emerging of compensation in donation with a focus on some known models of compensation for unrelated kidney donation as practiced by some countries.

## MATERIALS AND METHODS

A comprehensive search was made on Pubmed for studies, review papers and meta-analyses discussing different types of kidney donation, financially driven kidney transplantation and the ethics revolving around compensation. The inclusion criteria were based on the most relevant, most recent and most cited studies present in Pubmed. A summary table of the studies is presented in a Table-1.

**Table 1:** Summary of discussed studies.

Author and year of publication	Sample size (if present)	Findings
Ghods, 2009 (1)	Not applicable	Iran has a 20-year experience with a compensated and regulated living unrelated kidney donation program. This transplantation model was adopted in 1988 and was able to eliminate kidney transplant waiting list in 1999.
Wu & Fang, 2013 (2)	Not applicable	Financial compensation policy initiated in five pilot provinces and cities in China helped increase the concept of organ donation.
Ghahramani et al., 2013 (3)	Survey of 1280 nephrologists from 74 countries.	Thirty-seven percent agreed with the provision of free lifelong health insurance to donors. Forty-nine percent agreed with some form of compensation, and 26% agreed with direct financial compensation for living donors. Thirty-one percent believed that living unrelated donors should receive financial rewards, while 23% favored rewards to related donors. Twenty-seven percent were in favor of financial rewards for families of deceased donors.
Alkuwari et al., 2014 (4)	Not applicable	Hamad Medical Corporation initiated the Doha Donation Accord (DDA) in 2010 to develop deceased organ donation and live related kidney transplantation prohibiting trade in human organs and financial rewards for organ donation. It covers expenses throughout the whole process.
Chapman, 2018 (5)	Not applicable	A review paper discussing organ trafficking and transplant commercialism.
Akkina et al., 2011 (6)	Not applicable	A review discussing donor exchange programs.
Schweda & Schicktanz, 2009 (7)	Focus group discussions with 66 European citizens.	The group resisted organ commercialization. Many respondents stated that the altruistic form of donation is not a one-way relationship, but is based on mutual exchange.
van Buren et al., 2010 (8)	Survey of 250 living kidney donors.	Almost half of the respondents were in favor of financial compensation for living donors by the government. The majority of the living donors would not have wanted any financial reward for themselves, because they donated a kidney out of love for the recipient or altruistic principles.
Tushla et al., 2015 (9)	Not applicable	Consensus Conference on Best Practices in Live Kidney Donation took place in 2014. The following recommendations were established: (1) allocate resources for standardized reimbursement of living kidney donors’ lost wages and incidental costs; (2) pass legislation to offer employment and insurability protections to living kidney donors; (3) create an living kidney donor financial toolkit to provide standardized, vetted education to donors and providers about options to maximize donor coverage and minimize financial effect within the current climate and (4) promote further research to identify systemic barriers to living donation and living kidney donor transplantation to ensure the creation of mitigation strategies.
Mazaris et al., 2009 (10)	Survey completed by 108 medical and nursing staff in a Renal and Transplant center in London.	Live donor kidney transplant was considered ethically acceptable between blood relatives (100%), non-blood relatives and friends (92.6%) and strangers (47.2%). Around 34.3% believed there should be no financial reward, not even compensation for expenses, for donors; 8% favored direct financial rewards for donors known to recipients and 18% favored rewards for donors not known to recipients, while 57.4% of respondents supported compensation for expenses incurred for donors known to the recipient and 50.0% supported this kind of compensation when the donor was a stranger.
Mazaris et al., 2011 (11)	There were 464 participants (63.8% patients and 36.2% health-care professionals).	Around 80% were willing to donate to children, siblings, parents; around 70% to non-blood relatives or friends and around 15% to strangers. Around 50% were willing to receive a kidney from a stranger versus 80% from parents, siblings, children or relatives and friends. Around 29% did not approve financial reward for donors and 60% approved covering expenses for donors.
Peters et al., 2016 (12)	There were 1011 respondents from the US (427 males and 584 females).	Around 65% were willing to donate a kidney to anyone and around 59% were willing if a payment of $50,000 was made.
Kute et al., 2014 (13)	There were 56 patients and 140 KPDs in a single center in India between 2000 and 2013.	For the 56 KPD transplantations, graft survival was 97.5%. KPD was done to avoid blood group incompatibility (n = 52) or positive cross-match (n = 4).
Mierzejeweska et al., 2013 (14)	Not applicable	A review paper discussing improvement in transplant numbers in several countries that have adopted KPD.
Pham et al., 2017 (15)	Not applicable	A review discussing KPD and desensitization.
Kute et al., 2017 (16)	There were 3616 living donor kidney transplantations, 561 deceased donor kidney transplantations.	There were 300 transplants done by KPD in a single center in India between January 2000 and July 2016.
Catwell et al., 2015 (17)	Not applicable	A review paper discussing the four years’ experience of KPD in Australia.
Kute et al., 2017 (18)	There were 380 KPD transplantations.	There were 77 transplants done by KPD in a single center in India between 1 January, 2015 and 1 January, 2016. The reasons for KPD were ABO incompatibility (n = 45), sensitization (n = 26) and better matching (n = 6).
Ghods and Savaj, 2006 (19)	Not applicable	A review paper discussing the Iranian model which was adopted in 1998 to regulate and compensate living-unrelated donor renal transplant program and has helped decrease the number of patients on the waiting list.
Bailey et al., 2016 (20)	Semi-structured interviews with UK 32 deceased-donor kidney transplant recipients.	The following themes were identified for those who were against altruistic donation: Prioritizing other recipients above self; fear of acquiring an unknown donor's characteristics and concern for the donor for unnecessary risk. For those willing to accept a non-directed altruistic living donor kidney transplantation the following themes were identified: Prioritizing known above unknown persons, belief that they are as deserving as other potential recipients, and advantages of a living donor kidney transplantation.
de Castro, 2003 (21)	Not applicable	A review paper discussing commodification of human organs.
Ghods et al., 2001 (22)	There were 1000 patients in Iran (500 living unrelated donors and 500 recipients).	The majority of living unrelated donors (84%) were poor and no single wealthy individual was listed in the category.
Friedman, 2006 (23)	Not applicable	A review paper discussing the need to legalize payment for living organ donation to prevent exploitation of organs.

## DISCUSSION

The Middle East region features some insufficiencies in the transplantation mechanism. These comprise inadequate preventive medicine, uneven health infrastructure, and poor awareness in the general public and medical community about organ donation. Severe organ shortage and political instability have resulted in major unethical practices such as transplant tourism and organ trafficking (1), even though the Consensus Conference on Best Practices in Live Kidney Donation aimed to achieve financial neutrality for live kidney donations (9).

Several studies were conducted to poll the opinion of the general public, health professionals and patients in regards to this issue. Questionnaires completed by medical and nursing staff at West London Renal Transplant Center showed that the highest acceptable mode of donation reported by the participants was from blood related donors (100%) followed by non-blood relatives and friends (92.6%) and strangers (47.2%). Direct financial rewards were not considered an important motive by the majority of participants wherein a substantial minority favored direct financial returns (10). Another study exploring attitudes towards live kidney donation and commercialism was conducted among 1105 participants from health professionals and patients. Most participants accepted the idea of alternative types of donation while the minority (10%) of the participants found that commercialism is acceptable (11). An international survey investigated the attitudes and perceptions of nephrologists. The study showed that 49% of the physicians expressed agreeable attitudes towards some form of compensation with the higher number from the Middle East region, while 66% mentioned that financial rewards will contribute to an increase in living kidney donation (3). Another study in the Netherlands conducted on 250 donors showed that 20% would have wanted some forms of financial compensation for their donation and 47% wanted a decreased fee or a free health insurance premium (8). In a survey completed by 1011 participants in the United States (US), 68% were willing to donate and 59% would be more compelled to donate if a monetary sum was given (12).

Since the greatest obstacle in kidney transplantation is the limited availability of deceased / living kidney donors, all possible solutions to increase access to kidney transplant should be taken into consideration before resorting to paid donation. This has given rise to another type of donation known as kidney paired donation (KPD) that has been established to prevent commercial transplantation (13). It takes place when a potential kidney recipient who has a willing but incompatible live donor receives a kidney from the donor of another incompatible pair and vice versa (13). KPD programs have been established to increase availability of organs and to overcome several obstacles such as blood group incompatibility, tissue compatibility, highly sensitized recipients to donors, and improvement in transplant quality such as graft size and age difference (14). The first KPD took place in South Korea in 1991 (15). This model has been proven to be practical, legal, cost-saving and time-saving for facilitating living donor related transplant for patients who are incompatible with their healthy and willing living donor (13).

Numerous transplant centers have adopted this program with amounted increase in transplant numbers. For instance, in the US the number of paired kidney exchanges has increased from 2 in 2000 to 400 transplants annually (14). In India, there were 3616 living donor kidney donations and 561 deceased donor kidney donations in one center between January 2000 and July 2016, while 300 of these were through KPD. The success of the program was due to maintaining registry for incompatible pairs, good teamwork and counselling on KPD (16). In Australia, the KPD program was established in 2010 and the four years’ experience has proven to achieve a sufficiently large pool of donors and recipients to be able to find the best match. It also helped highly sensitized patients by combining KPD with antibody removing strategies (17). KPD was also proven to be beneficial to unpaired patients since by removing all patients with potential living donors from the waiting list, and it will decrease their waiting time (17).

The KPD program requires a national database that is absent in some countries like in India, in addition to lack of coordination between transplant centers (18). There are still inefficiencies in this system; high number of patients waiting on the list (more than 500 patients in one center in India). Another hurdle is that blood group O recipients had lower rate of transplantation and high mortality rate due to financial incapability to afford dialysis (13). The authors also discussed the issue of putting more pressure on women to donate since KPD eliminates the excuse of incompatibility as there is gender imbalance with women donating more than men and receiving less donations than men (13). Moreover, desensitization has arisen for highly sensitized patients such as patients in need for repeat kidney transplants (15). Although this technique involves plasmapheresis, it is costly and there is the risk of increase in antibody levels. However, there are not many studies discussing the outcome of desensitization (15).

The past decade has witnessed an upsurge in innovative mechanisms for living kidney donation such as altruistic donation, KPD, donor chains and exchange programs (3). However, these strategies, especially the last three were not capable of increasing the kidney donor pool nor capable of alleviating renal transplant waiting lists (19). As a result, altruistic donation may seem ideal for kidney transplantation, yet safety and ethical concerns remain. Living donors are at risks without any direct medical benefit to themselves (1) putting their safety and welfare at risk. Another note to take into account is whether these altruistic donors have health insurance and can maintain their health insurance following donation (3) or whether their medical expenses can be secured by another party (1). Thus, what if a healthy and altruistic individual is willing to donate but there is no available center that offers free medical tests or post-op follow-up for any complications, especially in developing countries? Altruistic donation provokes a feeling of indebtedness and guilt to the recipients (7). These recipients may be willing to discover who their donor is when sometimes the system does not allow them or they may express worries about the nature of the kidney donated or about the characteristics of the donor. A qualitative study examined the attitudes of 32 renal patients (17 females and 15 males) towards non-directed altruistic living donation (20). Concerns were raised over transmission of donor characteristics, feeling responsible for the risks donors would be exposed to and feeling guilty (20). Nevertheless, providing compensation for altruistic donors does not essentially lead to exploitation, but may help minimize the level of exploitation that already exists in current organ procurement systems (21).

If it is already taken for granted that financial rewards for organ donation are pernicious and accordingly prohibited by laws worldwide, why do these rewards still receive heightened attention? Why are they practiced clandestinely? What are the reasons behind the rise of compensation in kidney donation? Possible explanations include: surge of commercial kidney transplants even in the presence of strict rules against them, limited success of altruistic donations and incapability of governments to secure organs through organized networks. Only under exceptional conditions, does the human being exhibit willingness for uncompensated transfers and generosity to others? (19). Altruistic donation failed to eliminate severe organ shortage and lessen the number desperate recipients on the waiting list (21). These desperate patients refer to other means to secure a kidney in developing countries whether they are natives or coming from developed countries (1). Therefore, altruistic donation has not been successful in providing kidneys to those in need and in shortening the waiting period (21). In developing countries, altruistic living unrelated kidney donations are less commonly experienced and these donors are usually secured through commercial transplants (1). Kidney markets are regularly seen in developing countries, since dialysis is usually not funded by the governments and deceased donor kidney transplantation is scarce due to poor and inefficient procurement mechanisms (1). However, this does not mean that all kidneys that have been purchased go through unfair practices. Many recipients rely on more ‘negotiable’ and ‘democratic’ ways for receiving kidneys wherein they try to financially secure kidneys from their relatives, friends or colleagues without imposing serious or direct harms to these donors (19).

There is a major argument against compensation of living unrelated kidney donation. It revolves around the concept of commodification. This argument assumes that there are limits to what can be sold and since organs are valuable and priceless then this means that they cannot be sold. Once a kidney is put into marketplace, it denies the definite valuable organ of a human being and denies his / her dignity and worth (21). The argument, at first glance, may seem convincing, but many objections follow. It is true that organs in general should not be sold or purchased, but in most cases the individual who has made the choice of donation is actually doing so for a valid reason and humane excuse. No one can imagine the circumstances that force the person to make a vehement decision of giving up one of his / her kidney (21). These persons have probably no other means of securing money and the reasons would not necessarily include luxury goods. Most donors actually decide to sell their kidneys either to provide financial support to their families or to secure money for other reasons (21). Commercialization seems to be a win-win-situation wherein both the donor and recipient benefit (7) provided that the donor has been compensated fairly and has not been abused. If commercialization is prohibited, not only will the recipient be put at disadvantage, but also the donor who was willing to make some money. However, the recipient may still win by travelling to another country to look for another donor or by trying to pay the same donor furtively in the same center by pretending to be relatives or friends. This shows how difficult it is to stop commercialization even when it is prohibited by law in the host country since recipients may travel to other countries to secure kidney.

What would seem a good solution to end the malevolent practice of kidney trafficking? In an attempt to explore the reasons donors propel to sell their organs, a primary overall factor appears: poverty. To compensate for such unethical practices, adopting a regulated system incorporated with financial incentives can help eliminate commercial transplants. This can be done when KPD has failed for a specific donor. An example of such a successful program is the Iranian model, initiated in 1988 and governed by a charitable organization called the Dialysis and Transplant Patients Association (DATPA). The DATPA provides compensation for those who provide kidneys without the need for a broker or mediator since the government covers for the hospital expenses, immunosuppressive drugs and provides award and health insurance to the unrelated donor (19). As a result, the number of renal transplants performed increased noticeably in Iran and the renal transplant waiting list was completely eliminated in 1999 (1). Prior to this model, most patients in Iran used to travel to India to undergo paid transplantation and many of these transplants were associated with transmission of hepatitis and surgical complications (19). A controlled living unrelated donor renal transplant program was introduced in Iran in 1998 wherein volunteered living unrelated donors introduce themselves to the Dialysis and Transplant Patients Association without any need for middle men or agencies (22).

The concept of financial incentives was grouped in three categories. The first class focuses on the subject of compensation, wherein the donor is covered for the incurred detriments such as medical tests and examinations, health insurance, etc. This is important because many of the donors may not have insurance to cover for their tests and out-of-pocket expenses are linked to more extensive medical care in the long term (9). The second category is known as rewarded gifting that involves monetary or non-monetary form of appreciation without direct intention to encourage donation. Lastly, the market model is based on procurement and allocation of organs. Such a system will help control the existence of black markets, organ trafficking and coercive methods targeting underprivileged and economically disadvantaged persons (7). Donors should be competent enough to understand the risks and benefits of kidney donation and undergo psychological evaluation and sign an informed consent (1). In China, the Red Cross is the third party that is responsible for implementing the donation policy wherein they raise and manage funds for the financial compensation in a fair manner (2). This includes covering funeral expenses and financial assistance for the family of the donor in case the family was classified as a low-income family by the local Bureau of Civil Affairs (2).

What would be the benefits of a system that adopts a model similar to the Iranian model? Ghods elaborates this topic by addressing several concerns that could be raised by opponents of this type of donation. Patient and graft survival rates, as well as donor morbidity and mortality in Iran have comparable results when compared to conventional transplant centers in the country, since all donors are positively selected and screened for diseases (19). The Iranian model has not forced poor citizens to donate their kidneys due to its rewarding nature, as one study has indicated that among 500 participants, 50.4% of kidneys from paid donors were given to poor individuals (22). The model has not even eliminated the number of kidneys from deceased persons, as deceased-donor organ transplantation has increased steadily since its legislation from 1.8% in 2000 to 12% in 2004 and 2005 (19). As mentioned previously, this model also helped reduce the number of commercial or illegal transplants that used to take place prior to its introduction and the model also prohibits foreigners to benefit from this system (19). It is also important that donors display utmost understanding of the possible risks and complications of the surgery.

On the other hand, the Hamad Medical Corporation (HMC) has initiated the Doha Donation Accord (DDA) in 2010 which aims at meeting the needs for transplantation as to discourage patients from undergoing unsafe practices abroad (4). The DDA provides a broad health insurance for life for the donor, covers the whole incurred expenses and prioritizes the donor in case of end-stage organ failure. While this seems to be encouraging and has actually resulted in the reduction of transplant travel, the rise of recipients on the waiting list has increased remarkably from 21% in 2010 to 73% in 2014 (4). The National Living Donor Assistance Center (NLDAC) was initiated in the US in 2007 wherein donors were provided financial support to travel to the transplant center for eligible living donation (9). The program received 3918 applications until August 2013 and approved 89% of them and 74% of donors mentioned that they would have not donated without the support of NLDAC (9). The question remains whether the country is able to fulfill this high demand when solely relying on living related donors. Although the DDA seems to be more ethical than the Iranian model, it is inevitable to state that the DDA model is not applicable to most countries since they cannot afford to have such a system especially noting that Qatar is a wealthy country with a high Human Development Index.

If countries decide to adopt a model that provides financial reward to donors, payment should be governed by a balanced, objective and multidisciplinary body which determines standardized protocols for donors and recipients as well as a uniform fee (23). If countries are not ready to accept such a model due to cultural reasons or financial difficulties, it these rely heavily on raising awareness about organ donation. When asked about ways to encourage living donation, 50% of the 250 donors mentioned that the media plays a major role especially when living donors discuss their experiences to the public (8). Media can a play a robust role in encouraging deceased organ donation or altruistic organ donation.

## CONCLUSIONS

Kidney transplantation is currently the accepted mode of renal replacement therapy, which provides long-term and robust survival advantage (10). A drawback represents the shortage of organ availability whether cadaver or living. This has given rise to the implementation of other strategies, each with its own challenges. Unethical practices still take place in countries with refugees or poor and displaced people (23). Many centers are not even able to detect the secret planned financial reward wherein they pretend to be relatives in order to get accepted for transplantation. It is important to start considering financial compensation in order to protect the indigent donors and avoid organ trafficking. Thus, the idea of initiating governmental supervision with regulated compensation to living donors should be revisited, especially that no alternative solution is available until today (23).
